# Comparison between Sickle Cell Disease Patients and Healthy Donors: Untargeted Lipidomic Study of Erythrocytes

**DOI:** 10.3390/ijms24032529

**Published:** 2023-01-28

**Authors:** Husam B. R. Alabed, Paolo Gorello, Roberto Maria Pellegrino, Hovirag Lancioni, Roberta La Starza, Anna Aurora Taddei, Lorena Urbanelli, Sandra Buratta, Anair Graciela Lema Fernandez, Caterina Matteucci, Maurizio Caniglia, Francesco Arcioni, Cristina Mecucci, Carla Emiliani

**Affiliations:** 1 Department of Chemistry, Biology and Biotechnology, University of Perugia, 06100 Perugia, Italy; 2 Hematology and Bone Marrow Transplantation Unit, Laboratory of Molecular Medicine (CREO), Department of Medicine and Surgery, University of Perugia, 06132 Perugia, Italy; 3 Pediatric Oncology-Hematology, Azienda Ospedaliera di Perugia, 06100 Perugia, Italy; 4 Centro di Eccellenza sui Materiali Innovativi Nanostrutturati (CEMIN), University of Perugia, Via del Giochetto, 06123 Perugia, Italy

**Keywords:** mass spectrometry, lipidomics, untargeted metabolomics, sickle cell anemia, sickle cell disease, systems biology

## Abstract

Sickle cell disease (SCD) is one of the most common severe monogenic disorders in the world caused by a mutation on *HBB* gene and characterized by hemoglobin polymerization, erythrocyte rigidity, vaso-occlusion, chronic anemia, hemolysis, and vasculopathy. Recently, the scientific community has focused on the multiple genetic and clinical profiles of SCD. However, the lipid composition of sickle cells has received little attention in the literature. According to recent studies, changes in the lipid profile are strongly linked to several disorders. Therefore, the aim of this study is to dig deeper into lipidomic analysis of erythrocytes in order to highlight any variations between healthy and patient subjects. 241 lipid molecular species divided into 17 classes have been annotated and quantified. Lipidomic profiling of SCD patients showed that over 24% of total lipids were altered most of which are phospholipids. In-depth study of significant changes in lipid metabolism can give an indication of the enzymes and genes involved. In a systems biology scenario, these variations can be useful to improve the understanding of the biochemical basis of SCD and to try to make a score system that could be predictive for the severity of clinical manifestations.

## 1. Introduction

### 1.1. Sickle Cell Disease

Sickle cell disease (SCD), or sickle cell anemia, is an inherited hemoglobinopathy caused by a single amino acid substitution at the sixth residue of the beta (β)-globin subunit (p.Glu6Val), which results in the production of the characteristic Hemoglobin S (HbS) [1]. Hemoglobin S displays particular biochemical characteristics [2]: when HbS is deoxygenated, it undergoes crystallization with associated polymers that deform the structure of red blood cells (RBC) (the characteristic sickle shape). Cyclic deoxygenation is associated with a reduction in cell ion and water content (cell dehydration), increased RBC density, and further acceleration of HbS polymerization that make RBCs irreversibly sickled [3,4]. These sickle-shaped RBCs are rigid and dysfunctional, and they play a central role in acute and chronic clinical manifestations of SCD. First of all, the increased adhesiveness of the sickle cells causes microvascular obstructions in capillaries and small vessels, blocking blood flow with ischemic/reperfusion injury [4,5,6]. In addition, the presence of free hemoglobin (Hb) and free heme contributes to the local reduction in nitric oxide (NO) bioavailability and high levels of pro-oxidant and pro-inflammatory agents [4,6,7,8]. The increased RBC rigidity and the resultant reduced deformability cause either removal by macrophages (liver or spleen) or destruction within the circulation, leading to extra- and intra-vascular hemolytic anemia, respectively. Sickle cell disease is the most important hemoglobinopathy worldwide in terms of frequency and social impact, and it has recently been recognized as a global public health problem by the World Health Organization (WHO) and the United Nations (UN) [9]. It is estimated that approximately 300,000 newborns are affected each year, and that 75% of them are born in Sub-Saharan African regions where SCD is endemic [9,10,11]. It is also highly prevalent in India. Approximately 100,000 individuals are affected in the United States. The number of subjects affected in Europe is estimated to be between 20,000 and 25,000, but numbers are rising steadily in many countries, e.g., in northern Europe, because of recent migration [9,10,11,12]. In Europe, SCD is found in Southern Italy, the Balkans, and Greece [12]. However, over recent years, the number of affected subjects has increased to approximately 1900 [13] due to immigration from northern and central Africa, the Balkans, Central and South America, and the Far East [14]. Causes of death include infection, acute chest syndrome, stroke, renal failure, and pulmonary hypertension [15]. Vaso-occlusion, hemolytic anemia, and vasculopathy are the hallmarks of SCD. Other factors such as hypercoagulability and inflammation are also involved in organ damage caused by SCD. Despite being a monogenic disorder, SCD is a multisystem disease associated with organ damage due to either acute events (pain crisis, acute chest syndrome (ACS), stroke) or from subacute events in chronic SCD in progression (pulmonary hypertension; complications affecting multiple organs, including eyes and kidney) and shows phenotypic variability with a wide range of severe, and even life-threatening, consequences. Thus, SCD patient care is complex, and this is emerging as an important issue throughout Italy.

### 1.2. Lipidomics: Lipid Class, Molecular Species, and Lipid Building Block Analysis

Lipidomics, a branch of metabolomics, is a systems-based study of all lipids, the molecules with which they interact, and their function within the cell [16]. Recent research has discovered that any disruption of a biological system could alter the quantity and/or composition of that system’s lipid pool [17]. Furthermore, research has linked alterations in blood lipid profiles to the start of several important human diseases, including cardiovascular disease [18], hypertensive diseases [19], Alzheimer’s disease [20], and cancer [21,22]. As a result, lipid molecules have lately received a lot of interest in the research for biomarkers for diagnostic reasons. Therefore, lipidomics is today regarded as one of the most essential and fundamental “omics”, and it is a fast-emerging field of study [23]. Nowadays Mass Spectrometry/Liquid Chromatography (LC/MS) is the most often used approach in laboratories for studying the lipid profile [24]. This analytical technique makes it possible to quantitatively determine hundreds or thousands of individual lipid molecular species in each analysed sample. The complex data matrix produced in an analysis batch, in which dozens of samples are compared, is usually processed with bioinformatics tools such as LipidOne [25], Biopan [26], MetaboAnalist 5.0 web platform [27], or OmicsNet [28] to further study the changes in molecular species and their building blocks and associate them with their biochemical pathways.

### 1.3. Lipidomics of Erythrocytes in Other Studies

Regarding human erythrocytes, most research focuses on membrane phospholipids and their relationship to metabolic conditions and diet or disease [29,30,31]. A more limited amount of research has been carried out on the lipid composition of whole erythrocyte cells. In both cases, the lipid profile has been represented as the percentage composition of lipid classes, fatty acid profile of the lipid molecular species, or with a lipidomic approach that allows the study of all lipids’ molecular species.

At present, numerous studies, including those by Mikirova, N. et al. [32], Jakobik, V. et al. [33], Novgorodtseva, T. P. et al. [34], and many others, have gathered a lot of information on the composition of fatty acids obtained from erythrocytes or erythrocyte membranes. On the other hand, the scientific literature reports a very limited number of studies dealing with lipid classes inside the erythrocyte or whole erythrocyte lipidomics.

Nonetheless, highly interesting research on erythrocyte membrane phospholipids composition was published in 2017 by Aoun, M., et al. [32]. In this study, they demonstrated that phosphatidylethanolamines (PE) and phosphatidylcholines (PC), with respective values of 36.0 ± 4.40% and 31.8 ± 3.78%, were the two groups that showed the greatest consistency. Sphingomyelin (SM) accounts for 18.4 ± 2.37% of the total, phosphatidylserine (PS) for 9.38 ± 1.75%, and phosphatidylinositol (PI) for 4.47 ± 1.56%. These findings are mostly consistent with the information presented in the sixth edition of Lehninger’s Principles of Biochemistry [33].

Regarding the lipidomic approach of human erythrocytes, we were only able to find two works. The first one is by Leidl, K. et al. [34,35], who examined a variety of blood cells, including erythrocytes and their membranes. According to this study, the composition at the level of lipid classes was as follows: PC (29%), SM (28%), PS (18%), PE (17%), Ether-linked phosphatidylethanolamine (PE-O) (9%), Lysophophatidylcholine (LPC) (3%), Ceramides (Cer) (1.8%), and PI (1%). This study demonstrates also that there are differences in the lipid composition of erythrocyte membranes compared to the same cells. The second work by Loef, M. et al. [36], on the other hand, reports the inter-day reproducibility of lipid measurements with the Lipidyzer platform over a 6-week period in plasma and erythrocytes. They were able to identify and quantify 630 and 286 individual lipid species in plasma and erythrocytes, respectively.

### 1.4. Lipidomics of Erythrocytes of Patients with Sickle Cell Disease

The lipid composition of sickle cells has received little attention in the literature. To the best of our knowledge, only two works have been carried out using a lipidomic approach. Both concern the study of erythrocyte membranes and not whole cells.

The first, conducted by Connor W. et al. [37], compared the lipid profile of the erythrocyte membranes of sickle cell disease patients to those of healthy donors. They found that polyunsaturated acyl chains in PC and PE decreased considerably statistically, while saturated and monounsaturated acyl chains increased.

A second work is that conducted by Sanghani S. et al. [38]. They also described the lipid composition of erythrocyte membranes in sickle cell anemia. According to this study, LPC and PE decreased significantly compared to healthy persons, whereas SM and PC increased.

In the introduction, information on erythrocyte lipidomics was presented, which was found to be mainly limited and nearly totally absent in the case of sickle cell anemia erythrocytes. The reason why many studies have focused on erythrocyte membrane lipids is probably related to the absence of nucleus and mitochondria in human erythrocytes.

However, recent work by Song Y, et al. [39] has shown for the first time that plasma Free Fatty Acids (FFA) contribute to the synthesis of human erythrocyte triglycerides (TGs) in vivo, and that the fatty acid profile of erythrocyte TGs correlates with metabolic health. The authors point out that the accumulation of saturated fatty acids in erythrocyte TGs is in stark contrast to the highly unsaturated profile reported in erythrocyte membrane phospholipids.

Therefore, the goal of this study is to dig deeper into lipidomic analyses of erythrocytes and highlight any variations between healthy and patient subjects.

In fact, in the context of systems biology, any discrepancies may be attributed to clinical data, changes in the proteome, and, eventually, changes in the genome.

## 2. Results and Discussion

The lipidomic analysis allowed us to obtain a data matrix containing qualitative and semi-quantitative information of 241 lipid molecular species divided into 17 classes. Instrumental repeatability expressed as percentage of Relative Standard Deviation (RSD %) was measured on the three experimental replicates and ranged from 0.1% to 19.28% with an average of 7.18% (see Table S2). The following discussion is based on the analysis on the biological samples only.

Considering the complexity of the data produced, we proceed with the analysis of the qualitative and quantitative differences within lipid classes, then between molecular species, and finally between lipid building blocks.

### 2.1. Comparison within Class Lipid Profiles of Diseased and Healthy Red Blood Cells

The amount of each class is reported as sum of molecular species detected (annotated).

The results are expressed as mean and the standard error. HbS indicates the SCD patients with Hemoglobin S group while WT is Wild Type or Healthy donors’ group. The *p*-value of the comparison between these two groups was calculated. We also calculated the percentage amount of lipid classes among all analysed samples. The results are shown in Table 1.

The first observation from Table 1 is that the amount of total lipids in HbS samples is about 24% higher than in WTs (*p*-value < 0.024). This significant difference agrees with the results reported by Westerman M. et al. [40]. These researchers, using a different analysis technique, revealed a 19% increase in lipids in RBCs of sickle cell anemia patients.

As demonstrated in Table 1, the classes with the highest relative abundances are PC (29.634%), TG (24.761%), PE (PE and PE ether-linked) (17.185%), SM (11.229%), and PS (9.934%). 

On a qualitative level, these results are comparable to Rübsaamen’s thesis work [34]. With the exception of the TGs, the author of the thesis found that the major lipid classes present in human erythrocytes cells are PC, PE, PS, PI, SM, LPC, and Cer. Quantitatively, the relative abundances of some lipid classes are different. In contrast to Rübsaamen’s work, which found a percentage of 28% for SM among all lipid classes, this work reported a percentage of about 11%; another example is the LPC class, which is reported in this work as about 0.4% of all lipids, while in Rübsaamen’s work it is 3% of the lipid classes. Similarly, the percentage of the PS is about 10% in our work, while it is 18% in the other one. However, the quantities of PC, PE, PI, and Cer are very similar in both studies.

Although the presence of TGs in red blood cells may seem unusual (24.761%), it is consistent with Song Yilin et al.’s findings [39]. This group of researchers brought to light for the first time the presence of TGs within erythrocytes and its relationship to plasma TG. According to our analyses, CoQ10 is only found in the HbS samples. This information is consistent with what Niklowitz P. et al. [41] reported. They demonstrated that, when compared to erythrocytes from healthy persons, the content of CoQ10 in sickle cell anemia patients’ erythrocytes increased by a factor of two [41]. Given the absence of mitochondria in erythrocytes, the presence of acylcarnitine (CARs) inside these cells may seem odd. However, the research performed by Osorio J. et al. [42] supports this result and confirms the presence of carnitine and acetylcarnitine inside the erythrocytes and how the intra-erythrocyte acetylcarnitine has a significant relationship to the plasma levels. In our work we were able to confirm the presence of CARs (0.7278%), and we found a significant increase (*p*-value = 0.009) in sickle cell erythrocytes of HbS subjects. Overall, a statistically significant increase in specific lipid classes in HbS patients such as Cer, DG, PC, PE, and PS can also be noted. On the other hand, we were not able to detect other lipid classes such as platelet activating factor (PAF) and lysophosphatidylethanolamines (LPE); however, this could be the result of their absence or at least, their presence in concentrations below the limits of instrumental detectability.

With the data in Table 1, it was also possible to conduct additional studies on the interactions between the lipid classes using a well-known lipid class pathway [43,44,45]. The network transformation analysis was performed by studying the ratio of product/reagent weights for each sample. The average of the weights obtained in Hbs was compared to that of the WT samples to determine which reaction is activated or suppressed as indicated by A. Nguyen et al. [46]. This data cannot be directly deduced from the absolute abundances of the single lipid classes.

**Figure 1:**
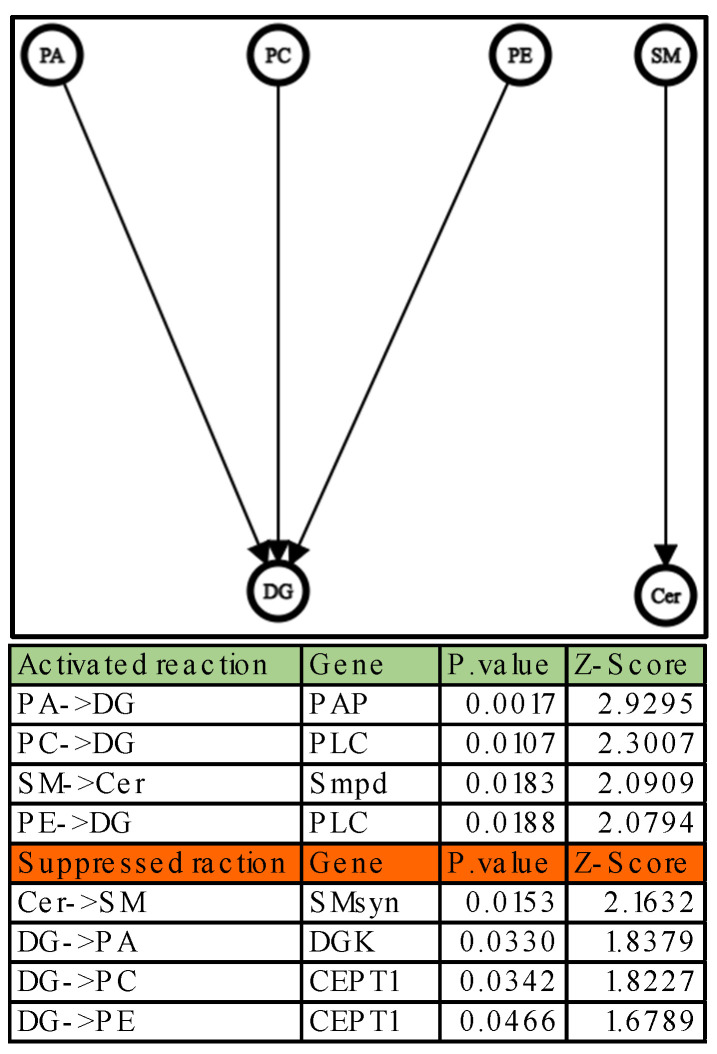
A network graph illustrates the possible transformations of lipid classes in the HbS group compared to WT group, which can be interpreted using known biochemical pathways as a reference. The graph was created with Graph Editor (https://csacademy.com/app/graph_editor/, accessed on 10 January 2023) using a statistical reprocessing of the data produced by LipidOne. The tables below show the activated and suppressed transformations, each with the possible gene involved, the associated *p*-value and Z-score.

As can be observed from Figure 1, most phospholipid classes (PA, PC, and PE) are being transformed into DG; the other transformation that can be observed is SM to Cer. It should be highlighted that all these transformations can be considered as a degradation towards less complex lipids. This formation of DG from other phospholipids can be the result of enhanced activation of phospholipase enzymes such as phospholipase C (PLC) and phosphatidate phosphatase (PAP) in HbS patients compared to WT [47]. However, it could be possible that there is a malfunction in the reverse pathway that transforms the DG into other phospholipids in the HbS patients. These pathways are carried out by a group of phosphotransferase enzymes such as choline/ethanolamine phosphotransferase 1 (CEPT1) and diacylglycerol kinase (DGK). These irregular activities in pathways could explain the significant increase in DG in HbS patients. In fact, many studies have shown that an altered concentration of DG has serious effects associated with diseases such as cancer, diabetes, immune system disorders, and Alzheimer’s disease [48]; other studies have reported the ability of DG to modify the activity of membrane-bound enzymes such as protein kinase C and phospholipases, which are essential for the metabolism of other lipids [49].

On the other hand, an enhanced activation of SM degradation in the HbS samples compared to WT can be noted. Some sphingolipids are bioactive and participate in intracellular signaling mechanisms, as demonstrated by Schneider-Schaulies S. et al. [50] in a recent publication. In particular, the breakdown of SM in Cer through the activities of sphingomyelinases (Smpd) or other specialized hydrolases triggers the formation of sphingosine 1-phosphate (S1P), a powerful signaling molecule. 

### 2.2. Analysis at the Level of Lipid Molecular Species

**Figure 2:**
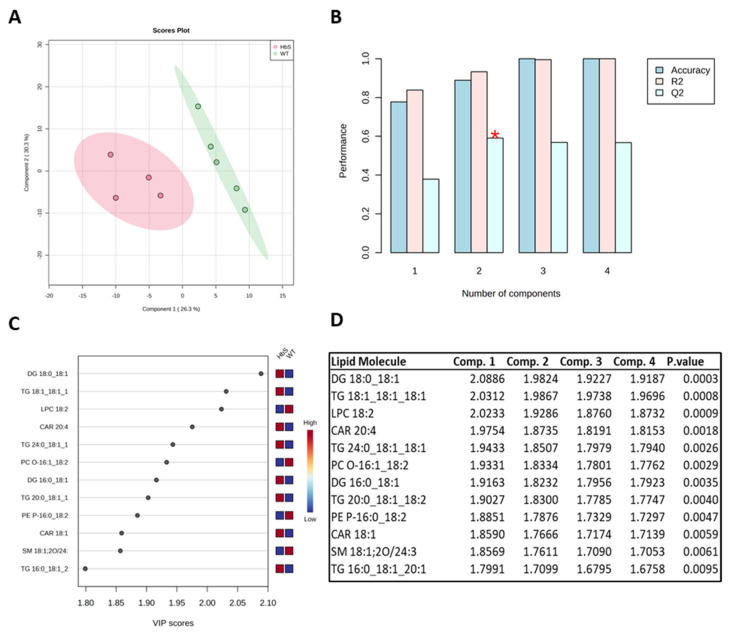
Multivariate statistical analysis of lipid species in HbS and WT erythrocytes: (**A**) PLS-DA. (**B**) Cross Validation test. (**C**) VIP (Variable Importance in Projection) plot; the colored boxes on the right indicate the relative concentrations of the corresponding lipid in each group under study. (**D**) Table of the VIP score in each component and *p*-values for the individual lipids in the VIP plot.

A supervised multivariate chemometric statistical method, such as Partial Last Squares Discriminant Analysis (PLS-DA), can be used to examine the data matrix that provides the concentration of 241 lipid species in each of the biological samples analysed. The statistical analysis shown in Figure 2 was carried out using the MetaboAnalist web platform after the normalization of the data matrix by median and scaled with the autoscaling algorithm option offered in the platform. The ellipses enclose the scores inside a region with a 95% confidence.

The two groups are easily distinguished using both the first (26.3%) and second (30.3%) components of the supervised statistical method PLS-DA (Figure 2A). A cross validation test has been performed to calculate the Q2 score of the PLS-DA (Q2 in 2 components = 0.5904 and R2 in 2 components = 0.9327) (Figure 2B). The gap between these two values indicates a non-excellent predictive ability of the model. The two clusters in the PLS analysis were mostly formed by the 12 lipid molecular species with the best *p*-value, which are seen in Figure 2C,D. The diagram demonstrates that three phospholipids (LPC 18:2, PC-O 16:1_18:2, and PE P-16:0_18:2) and one sphingomyelin (SM 18:1;2O/24:2) are under-expressed in HbS. However, HbS samples present higher levels of six glycerolipids (DG 18:0_18:1, TG 18:1_18:1_18:1, TG 24:0_18:1_18:1, DG 16:0_18:1, TG 20:0_18:1_18:2, and TG 16:0_18:1_20:1) and two acylcarnitines (CAR 20:4, and CAR 18:1) compared to WT samples.

**Figure 3:**
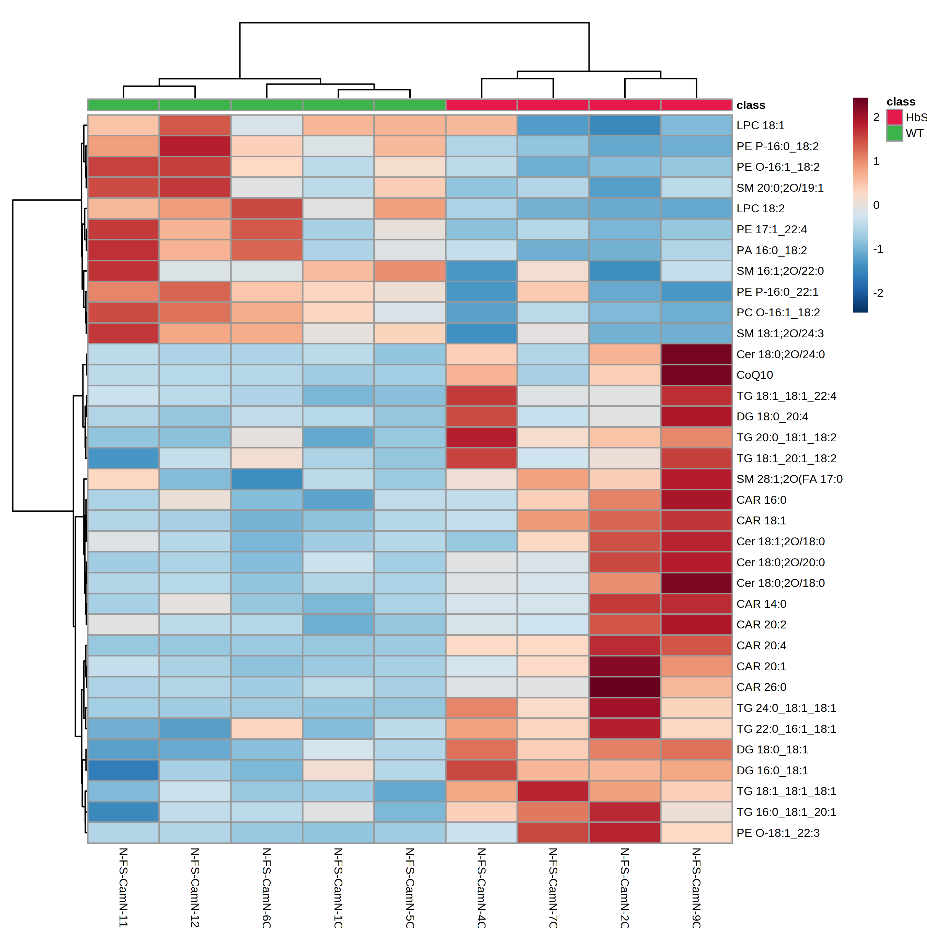
Heatmap: The figure shows the correlation of the 35 most significant lipid molecular species within each sample. The color shows the level of expression of each individual species in each sample based on their concentrations. The analysis was performed using the Pearson correlation index and the complete clustering method.

The heatmap in Figure 3 shows that some molecules species of CAR, TG, DG, Cer, and CoQ10 are over-expressed in the HbS group; however, several phospholipid and SM species are under-expressed.

### 2.3. Analysis of Lipid Building Blocks

LipidOne was also used to perform in-depth analysis of lipid building blocks inside the lipids of the two experimental groups.

**Figure 4:**
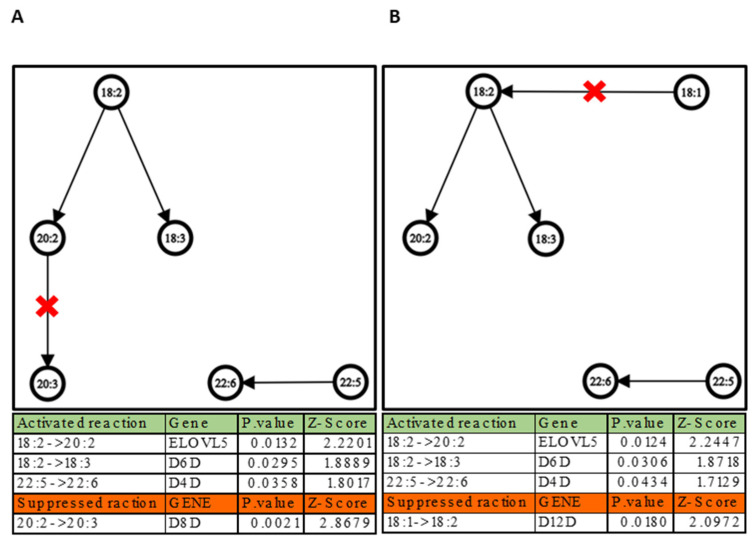
A network graph depicts the interconversion of the building blocks in the HbS group compared to the WT at the chain length level and unsaturation level of fatty acids inside the lipid molecules. (**A**) The interconversion of the building blocks in the HbS group compared to the WT inside all 17 lipid classes. (**B**) The interconversion of the building blocks in the HbS group compared to the WT inside the phospholipid classes (LPC, PA, PC, PE, PI and PS). The table below each graph demonstrates the possible activated/suppressed reaction with their *p*-value and z-score and the genes that could be involved in the interconversion.

As mentioned earlier, LipidOne examines lipid building components, highlighting the length of chains and the amount of unsaturation within each lipid class, and calculating their semi-quantitative presence. The network graph in Figure 4 depicts the result. It should be noticed that the interactions and interconversion within the chains in HbS inside all the 17 lipid classes (Figure 4A) and inside only the phospholipid classes (Figure 4B) are similar, which could mean that the changes inside the building blocks are happening mostly within the phospholipids. The figure demonstrates that the interconversion within the chains in HbS are geared toward the synthesis of chains with 20 carbon atoms (FA 20), through elongation of fatty acids with 18 carbon atoms. These modifications point to a significant activation of the chain-elongation process. This process could be performed with a group of enzymes known as fatty acid elongase (ELOVL), specifically ELOVL fatty acid elongase 5 (ELOVL5) [45]. Other significant interconversions among building blocks shown in the figure are the desaturation of the fatty acids. It can be noted that there is a significant increase of specific chains with multiple unsaturation such as FA 18:3 and FA 22:6 formed by the desaturation of FA 18:2 and FA 22:5, respectively. This behavior could suggest an increase of the activation of desaturase enzymes such as delta-6 and delta-4 desaturase inside the HbS group. On the other hand, the study also suggests a decrease in the activation/suppression of other desaturates such as delta-8 and delta-12 desaturates in the HbS group, which are responsible of the desaturation of FA 20:2 to FA 20:3 and FA 18:1 to 18:2, respectively.

**Figure 5:**
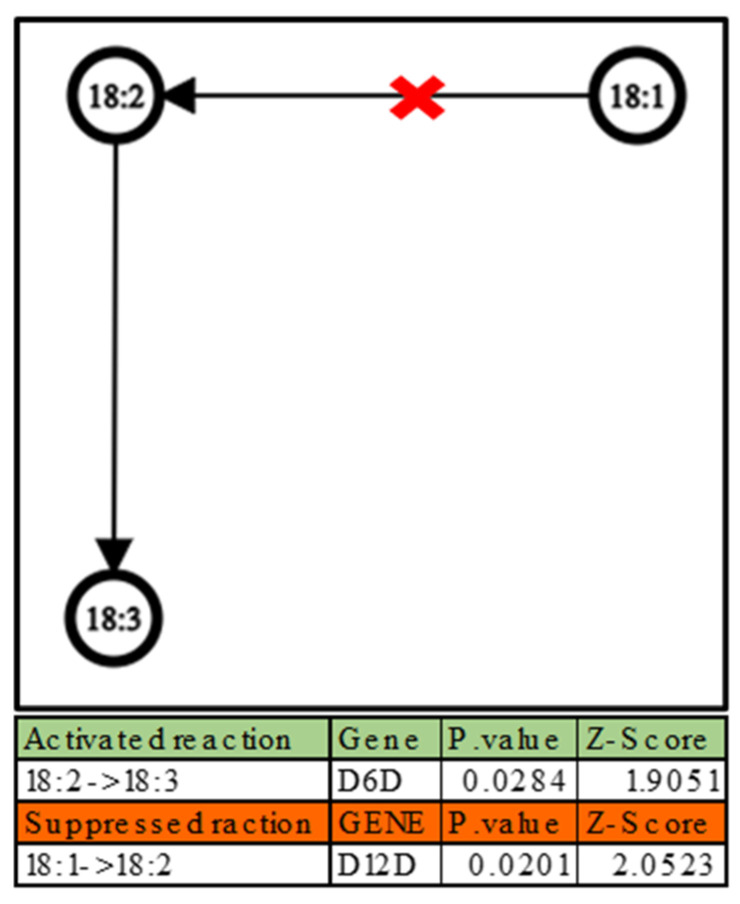
Network graphs depict the interconversion of the building blocks in the PCs’ lipid class in the HbS group compared to the WT. The table below the graph demonstrates the possible activated/suppressed reactions with their *p*-values and Z-scores and the genes that could be involved in the interconversion.

Since PCs represent over 29% of the total lipid fraction, a building block study was also carried out for this phospholipid class. Results are summarized in Figure 5.

Figure 5 shows a similar behavior in terms of chain unsaturation, indicating that the interconversion between the chains results in an increase of formation of chains with 18 carbon atoms with 3 unsaturated and decrease of the formation of chains with 18 carbon atoms with 2 unsaturated inside the HbS group. This behavior suggests an altered regulation of desaturase enzymes such as delta-6D, which is responsible for the desaturation of FA 18:2, and delta-12D, which is responsible of the desaturation of FA 18:1 [45].

## 3. Materials and Methods

### 3.1. Chemicals and Reagents

LC/MS (Merck KGaA, Darmstadt, Germany) grade Water (LiChrosolv), Acetonitrile (ACN), Methanol (MeOH), and Isopropanol (IPA) all (Carlo Erba, Milan, Italy), ammonium fluoride, ammonium acetate, methyl-t-butyl ether (MTBE), and chloroform (Chlo) were purchased from Sigma-Aldrich (Sigma-Aldrich GmbH, Hamburg, Germany). Internal standard (IS) was Splash Lipidomix (Avanti Polar, Alabaster, AL, USA): mixture with known concentration of the following lipids [nmol/mL]: PC 15:0_18:1(d7) [212.6]; PE 15:0_18:1(d7) [7.0]; PS 15:0_18:1(d7) [6.6]; PG 15:0_18:1(d7) [40.5]; PI 15:0_18:1(d7) [12.1]; PA 15:0_18:1(d7) [10.5]; LPC 18:1(d7) [47.3]; LPE 18:1(d7) [10.3]; CE 18:1(d7) [532.2]; MG 18:1(d7) [5.5]; DG 15:0_18:1(d7) [17.0]; TG 15:0_18:1(d7)_15:0 [67.8]; SM 18:1;2O/18:1(d9) [40.7]; Cholesterol(d7)[254.2].

### 3.2. Samples

The University of Perugia’s Department of Medicine and Surgery has made 10 blood samples accessible, five from healthy subjects, WT (Wild Type), and four from SCD patients. Genotyping was performed on the HBB gene, and the mitochondrial control region was sequenced as previously reported in Barbanera Y. et al. [51]. Patients resulted homozygous for hemoglobin S (HbS) and showed different mitochondrial haplotypes classified into four haplogroups. Table 2 shows genetic and clinical data that were collected for each subject. 

### 3.3. Preparation and Storage

Blood was obtained in anticoagulant-containing tubes. The red blood cells were subsequently purified using the technique reported in Breitling-Utzmann CM et al.’s [52] work with minor changes. The samples were centrifuged to separate the corpuscular portion of the blood from the plasma. The white blood cells visible at the interface were then eliminated along with the plasma. The red blood cells were resuspended in an equivalent amount of Phosphate-Buffered Saline (PBS) and washed three times. During the final wash, the erythrocytes were counted and aliquoted in separate Eppendorf tubes to yield 2 × 10^9^ cell pellets. The pellets were kept at −80 °C.

### 3.4. Preparation of Analytical Samples and Lipid Extraction

Three 5 × 10^6^ erythrocyte pellets (experimental replicates) were collected from each biological sample conserved from the previous step, for a total of 27 experimental samples, 15 of which were healthy (WT) and 12 of which were sick (HbS). The experimental replicates were used to verify the Relative Standard Deviation (RSD) (See Table S2). With slight modifications, lipid extraction was performed on the pellets following the technique reported by Pellegrino et al. [23]. Firstly, a 30 mL MMC (MTBE, MeOH, and Chlo) extraction mixture was created by adding 1 mL of (IS) diluted 1:10 in MeOH, to 9 mL of MeOH, 10 mL of MTBE, and 10 mL of Chlo. Afterwards, 2 mL of the MMC mixture was added to each sample. After 30 s of vortexing, the samples were immersed in an ultrasonic bath until a cell aggregation formed destroyed. Following further 30 s of vortexing, the samples were placed in a T-Shaker (Euroclone, Milan, Italy) and treated for 20 min at 1500 rpm at 20 °C. Following that, the samples were centrifuged at 16,000× *g* for 10 min at 4 °C, and the supernatant was transferred to an autosampler vial. The same procedures were followed on a pool of all samples as well as an Eppendorf containing only the solubilization solution (MMC), which was later used as a blank.

### 3.5. LC/MS Analysis

Agilent analyzer, which consists of the Agilent 1260 Infinity II liquid chromatograph coupled with the Agilent 6530 Accurate-Mass Q-TOF (Quadrupole-Time of Flight) analyzer and Agilent JetStream source, was used for the LC/MS analyses. The chromatographic separation of the lipids was carried out by a reverse phase C18 column (Agilent C18 Poroshell column with the dimensions of 100 × 3 mm and particle diameter 2.7 μm) maintained at 60 °C and 0.6 mL/min flow. The mobile phase consisted of a quaternary gradient of water (solvent A), (ACN) (solvent B), (MeOH) (solvent C), and (IPA) (solvent D). All solvents were added to ammonium fluoride at a concentration of 0.2 mM. IPA, MeOH, and water were also prepared with ammonium acetate at a concentration of 10 mM. The gradient was as follows: the time 0–1 min had an isocratic gradient at A 27%, B 14%, C 24%, and D 35%; the time 1–3.5 min had a linear gradient to A 12.6%, B 17.2%, C 27.2%, and D 43%; the time 3.5–10 min had a linear gradient to A 0%, B 20%, C 30%, and D 50%; the time 11–17 min had a linear gradient to A 27%, B 14%, C 24%, and D 35%; and the time 19 min: stop run.

In full scan mode, spectrometric data were collected in the 40–1700 m/z range for both negative and positive polarity. The pool sample was analysed five times in an iterative DDA acquisition mode to gather the most MS/MS possible spectra. The Agilent JetStream source ran on the following protocol: 200 °C for the N2 gas, 10 L/min for the drying gas, 50 psi for the nebulizer, and 300 °C at 12 L/min for the sheath gas. (See Figures S1–S3 for examples of chromatographic separation and tandem mass spectrometry data in both ionization modes.)

### 3.6. Raw Data Processing

Peak-picking, alignment, annotation, semi-quantification, and polarity amalgamation were performed on the raw data using the open-source software MS-DIAL (4.48) [53].

Lipid annotation at the molecular species level was performed using MS and MS/MS data in accordance with the Lipid Standard Initiative’s (LSI) recommendations [54]. Following the procedure indicated by Tsugawa H. et al. [53], lipid semi-quantification was performed using the deuterated internal standard (Splash Lipidomix) for each lipid class at levels 2 and 3 of LSI recommendations (See Tables S1 and S3).

A data matrix with the concentration in nmol/50 million red blood cells of 241 annotated lipids spread across 17 lipid classes was obtained at the end of the operation. LipidOne [25] was used for in-depth analysis of lipid building blocks, and the MetaboAnalist 5.0 web platform [27] was used to process this dataset for multivariate statistical and chemoinformatic analysis. By analyzing the data received with LipidOne, network graphs were produced using Microsoft Excel and Graph Editor (https://csacademy.com/app/graph_editor/, accessed on 10 January 2023).

## 4. Limitations

This study is exploratory in nature. It has two main limitations. The first is certainly the small sample size analysed, due to the difficulty in obtaining relatively young donor patients. This limitation did not allow for the investigation of potentially confounding aspects that may influence the erythrocyte lipid profile, such as age, sex, diet, and body mass index of the donors (both healthy and sick).

A second limitation is that the untargeted lipidomic approach revealed the presence of some unexpected lipid classes and subclasses, not represented by corresponding labelled species in the mixture of internal standards used (Splash Lipidomix). This could have caused quantitative over- or under-assessment of some minor lipid classes/subclasses (such as CAR, CoQ, ST, PC-O, and PE-O).

## 5. Conclusions

Despite the limitations, the results of the study allowed us to draw the following conclusions: after analysing the lipid profiles of red blood cells of healthy subjects and SCD patients, we were able to annotate 241 lipid species divided into 17 classes. Numerous studies only discuss the erythrocyte membranes’ lipid content. The only published study on the lipidomic analysis of entire erythrocytes appears to be in conflict with the relative abundance of various lipid groups found by us. However, the sample preparation and the lipid extraction method could be one of the reasons for these differences. 

In addition to phospholipids, sphingomyelins, and ceramides, lipids from unexpected classes including TGs, CoQ10, and CARs were also highlighted. In other works, even in non-lipidomics ones, diverse authors have verified that each of them is present inside of these cells. Moreover, according to the statistical analysis, there are significant differences between lipid species that belong to various classes. We can confirm that certain compounds from the classes of CARs, TGs, DG, Cer, and CoQ10 are over-expressed in the samples of sickle cell disease patients compared to healthy subjects, and certain phospholipid and SM species are under-expressed. Furthermore, the data from the analysis of the lipid building blocks show that there is an enhanced activation for the synthesis of fatty acids with 20 carbon atoms from fatty acids with 18 carbon atoms. This analysis also shows that there is an enrichment of phospholipid species, especially PC-containing fatty acids with multiple unsaturations.

The information acquired allows us to reach a key conclusion: subjects with SCD have erythrocytes with a different lipid profile compared to healthy subjects. The changes that take place on specific molecular species that correspond to various lipid class, such as PC, PE, and SM, which were noted to be lower in the patients, as well as an accumulation of other lipid groups such as TG, DG, CARs, Cer, and CoQ10, can suggest significant changes in cellular metabolism within these two groups. These changes in the lipid profile may be reflected in the erythrocyte membrane biology and behavior in HbS patients, for example, the increase in DG concentration in patients’ erythrocytes, which would appear to be due to irregular activation of PLC, PAP, or DGK, could lead to a disruption of the lamellar phase of lipid membranes [55]. 

Other studies have reported a link between DG and the concentration of calcium ions inside cells. A change in the concentrations of DG could cause an alteration of the concentration of calcium ions in the cells, which in turn could affect the cell cycle and the fate, metabolism, structural integrity, motility, and cellular volume. In fact, an excessive accumulation of calcium ions has been associated with a number of diseases including SCD [56,57,58].

Several studies suggest that PS exposure could increase deoxygenation, hemoglobin polymerization, and sickling shape change [59]. This could be explained by the significant increase of the concentration of PS inside the erythrocytes of the HbS patients.

Another aspect that has been highlighted in this study is the alteration in the building blocks of lipids (the length of the fatty acids and their unsaturation). In fact, even if the presence of unsaturated fatty acids is essential for the fluidity of the cell membrane, an excessive increase of these fatty acids could destabilize the membrane. On the other hand, the presence of longer fatty acids causes a lower fluidity and permeability of the cell membrane. All these signals could suggest a serious alteration of the cell membrane in terms of flexibility causing the cell membrane of the patients’ erythrocytes to become more rigid, which in turn favors the aggregation of sickle cells. 

The relevant metabolic pathways and the malfunctioning enzymes can all be identified by extrapolating these statistically significant changes onto cellular metabolism. These changes could be caused by alteration of the activity of some enzymes we mentioned before, such as some elongases, desaturases, phospholipases, and sphingomyelinases. In a systems biology scenario, these variations can be useful in order to improve the understanding of the biochemical basis of SCD. Since the patients have the same genetic profile (genotype HbS/HbS) with various mitochondrial haplogroups, this study can allow possibilities to make a score system that could be predictive for the severity of clinical manifestations and could refer the patient to the treatment that may be most suitable, given that many new drugs and new targets are emerging for SCD. The indications obtained in this study are interesting; however, to corroborate the data discovered and improve the significance of statistical tests, it will be essential to recruit additional patients in the future. The effects of age and gender and other variables known to influence lipid profile will also need to be explored.

## Figures and Tables

**Table 1:** Comparison of the concentration of lipid class of human erythrocytes cells in the two experimental groups HbS (n = 4) and WT (n = 5). The average concentrations are reported in nmol/50 million of RBC. The number of annotated molecules is the number of single molecular species revealed.

Lipid Class Abbreviation	Explained Class Name	Number of Annotated Molecules	Average ± Exp Er (WT)	Average ± Exp Er (HbS)	*p*-Value	% Amount
CAR	Acylcarnitine	13	0.26 (±0.033)	1.031 (±0.285)	0.009	0.728
CE	Cholesteryl ester	4	3.036 (±0.612)	1.653 (±0.246)	0.049	2.642
Cer	Ceramide	13	0.994 (±0.095)	2.297 (±0.542)	0.016	1.854
CoQ10	Coenzyme Q	1	0.00 (±0)	0.005 (±0.002)	0.020	0.003
DG	Diacylglycerol	13	0.043 (±0.008)	0.117 (±0.024)	0.007	0.090
LPC	Lysophophatidylcholine	6	0.291 (±0.018)	0.348 (±0.061)	0.179	0.360
PA	Phosphatidic acid	4	0.589 (±0.038)	0.623 (±0.086)	0.355	0.682
PC	Phosphatidylcholine	21	22.378 (±1.034)	30.224 (±3.625)	0.027	29.634
EtherPC (PC-O)	Ether-linked phosphatidylcholine	3	0.176 (±0.012)	0.294 (±0.077)	0.065	0.265
PE	Phosphatidylethanolamine	20	4.905 (±0.225)	7.016 (±0.93)	0.021	6.716
EtherPE (PE-O)	Alkyl Ether-linked phosphatidylethanolamine	18	4.9 (±0.22)	6.602 (±0.909)	0.041	6.480
EtherPE (PE-P)	Vinyl Ether-linked phosphatidylethanolamine	10	3.144 (±0.183)	3.937 (±0.448)	0.059	3.989
PI	Phosphatidylinositol	7	0.418 (±0.053)	0.633 (±0.1)	0.042	0.592
PS	Phosphatidylserine	12	7.224 (±0.428)	10.409 (±1.581)	0.034	9.934
SM	Sphingomyelin	28	8.896 (±0.386)	11.036 (±1.167)	0.048	11.229
ST	Sterols	2	0.044 (±0.012)	0.031 (±0.011)	0.227	0.042
TG	Triacylglycerol	66	18.965 (±5.395)	24.987 (±4.574)	0.219	24.761
Total		241	76.264 (±5.629)	101.242 (±9.374)	0.024	100.000

**Table 2:** The table shows genetic and clinical profiles acquired for each sample: the age, the number of red blood cells for microliter (RBC/uL), the level of hemoglobin present (Hb), the value of MCV (Mean Corpuscular Volume), MCH (Mean Cell Hemoglobin), MCHC (Mean Corpuscular Hemoglobin Concentration), mitochondrial haplogroups (HG), and the number of days since the last transfusion for the HbS subjects.

Samples ID	Genotype	Mitochondrial HG	Age	×10^−6^ RBC/µL	Hb (g/dL)	MCV (fl)	MCH (pg)	MCHC (g/dL)	Transfusion (Days)
2	HbS/HbS	T1a1+@152	13	3.28	8.7	81.9	28.2	34.4	96
4	HbS/HbS	L3e1b2	17	3.16	9.7	89.1	32.1	36.1	42
7	HbS/HbS	L3e4a	3	3.87	9.3	73.9	26.6	36	120
8	HbS/HbS	H34*	9	2.48	8.1	93.2	34.2	36.7	43
1	wt/wt	-	6	3.7	12.4	95.5	32.9	34.4	NO transfusion
5	wt/wt	-	6	4.6	14.1	88.2	32.5	36.8	NO transfusion
6	wt/wt	-	8	4.95	12.2	74.6	25.2	33.7	NO transfusion
11	wt/wt	-	18	4.93	14.4	86.3	29.9	34.7	NO transfusion
12	wt/wt	-	5	4.78	13.5	82.6	29	35.2	NO transfusion

## Data Availability

The datasets generated during and/or analysed during the current study are available from the corresponding author on reasonable request.

## Supplementary Materials

The following supporting information can be downloaded at: https://www.mdpi.com/article/10.3390/ijms24032529/s1.
