# Supplementary material for: Comparison between Sickle Cell Disease Patients and Healthy Donors: Untargeted Lipidomic Study of Erythrocytes
Source: Int J Mol Sci. 2023 Jan 28;24(3):2529. doi: 10.3390/ijms24032529 (PMC9917006; doi:10.3390/ijms24032529)
Supplement: Supplementary file 1 [file ijms-24-02529-s001.zip › Supplementary Materials-IJMS Lipidomic Sickle Cells Figures S1-S3.pdf]

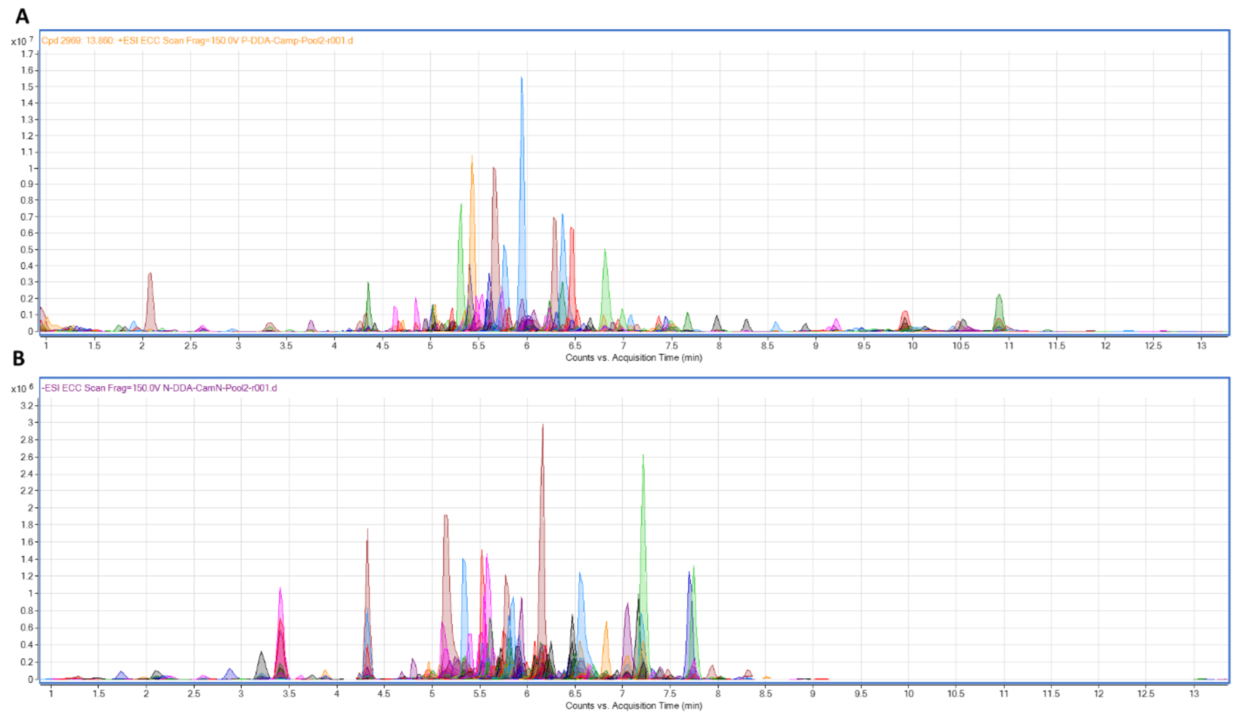

Figure S1: Example of chromatogram obtained by the algorithm "Find Compounds by Molecular Feature" of Mass Hunter Agilent software in both ionization modes. **A**) Positive ions (+H, +NH4). **B**) Negative ions (-H, +CH3COO).

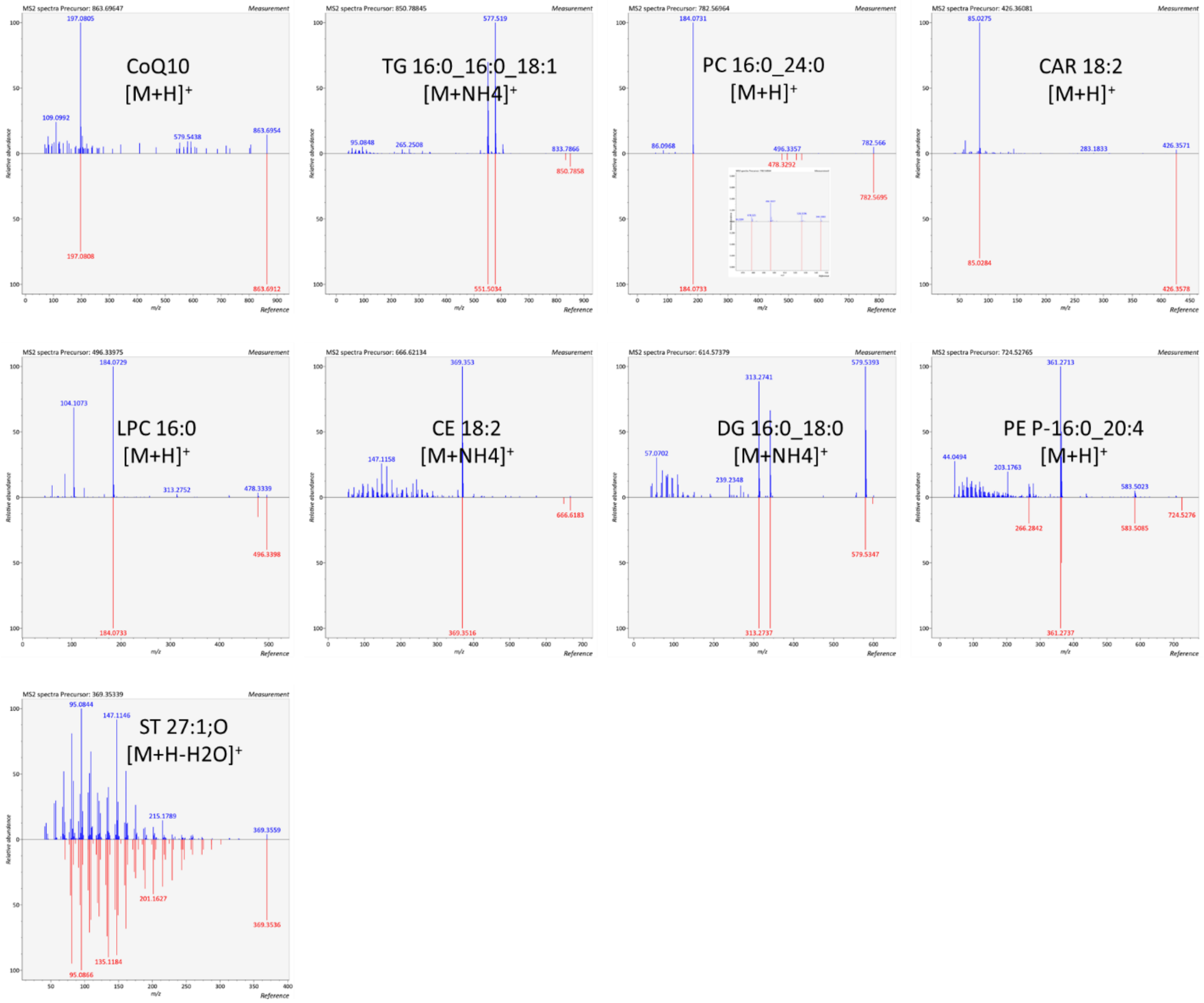

Figure S2: Example of comparation between the experimental MS/MS spectra (blue) and the reference MS/MS spectra (red) from LipidBlast library for each lipid class in positive ion mode.

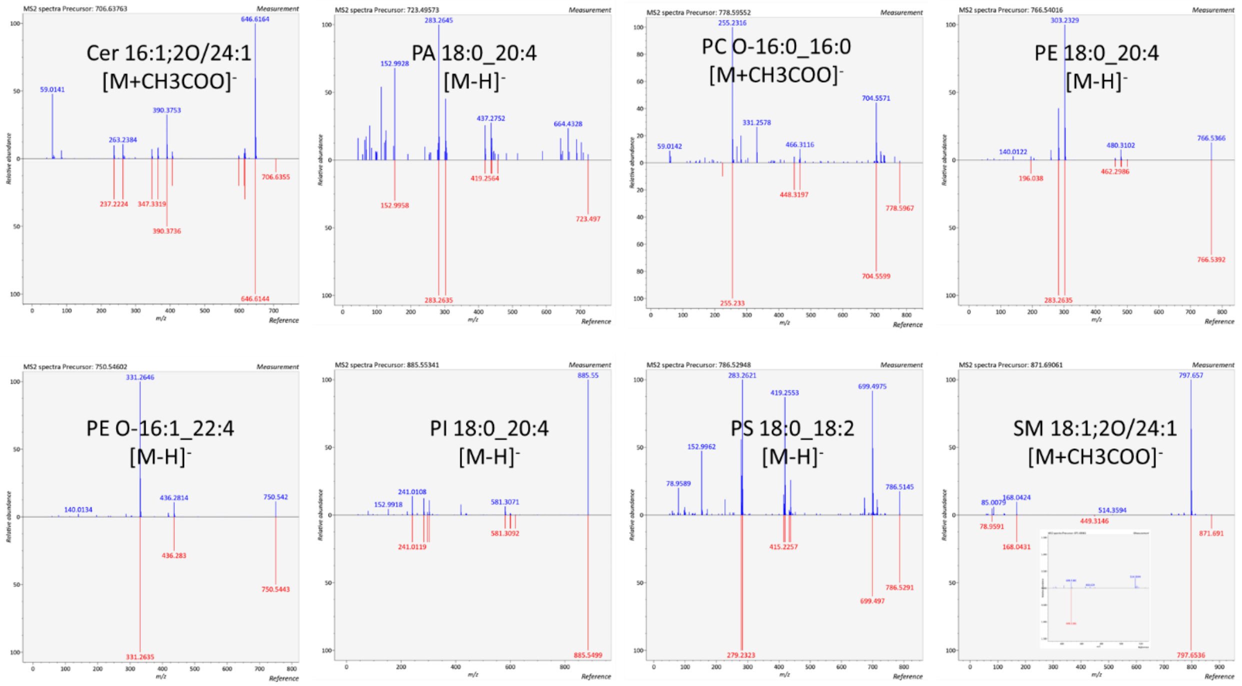

Figure S3: Example of comparation between the experimental MS/MS spectra (blue) and the reference MS/MS spectra (red) from LipidBlast library for each lipid class in negative ion mode.
